# Front-line decision-making: a thematic analysis of interviews with hospital staff on referrals, admissions, and care for people with multiple long-term conditions

**DOI:** 10.1186/s12916-026-05236-6

**Published:** 2026-10-16

**Authors:** Sara Pretorius, Sue Bellass, Rachel Cooper, Felicity Evison, Suzy Gallier, Nicola Howe, Elizabeth Sapey, Amelia Sheppard, Jana Suklan, Avan A Sayer, Miles D Witham, on behalf of the ADMISSION Research Collaborative

**Affiliations:** 1 NIHR HealthTech Research Centre in Diagnostic and Technology Evaluation, NE2 4HH Newcastle upon Tyne, England, UK; 2 AGE Research Group, Translational and Clinical Research Institute, Faculty of Medical Sciences, Newcastle University, Newcastle upon Tyne, England, UK; 3 NIHR Biomedical Research Centre (BRC): Newcastle, Newcastle upon Tyne, England, UK, Newcastle upon Tyne, England, UK; 4 PIONEER, The HDRUK Data Hub in acute care, University of Birmingham, Birmingham, England, UK; 5 Data Science Team, Research, Development & Innovation, University Hospitals Birmingham NHS Foundation Trust, Birmingham, England, UK; 6 Institute of Inflammation and Ageing, University of Birmingham, Birmingham, England, UK

**Keywords:** Multiple long-term conditions, Multimorbidity, Clinical decision-making, Hospital referrals, Hospital admissions, Care pathways, Patient care, patient management

## Abstract

**Background:**

Multiple long-term conditions (MLTC) are increasingly common and present challenges for hospital healthcare professionals (HCPs) making decisions about admissions, referrals, and care management in the context of a single-organ oriented health system. This qualitative study examined how HCPs in emergency and acute hospital settings make such decisions for people living with MLTC, exploring factors that influence clinical judgement and how HCPs respond to these challenges.

**Methods:**

HCPs involved in making decisions about care for people living with MLTC presenting to hospital were recruited via National Health Service (NHS) research delivery networks and professional contacts, using purposive and snowball sampling. Semi-structured individual interviews were conducted, focusing on how HCPs make decisions regarding the care of people living with MLTC and factors influencing such decisions. Reflexive thematic analysis was undertaken. Themes relating to influences on decision-making were organised using the Social Ecological Model (SEM).

**Results:**

We interviewed 40 HCPs working in NHS emergency departments and acute assessment units across multiple regions, specialties and roles. Seven themes were constructed and organised into two overarching categories. The first category ‘Factors Influencing Decision-Making’ included the themes *the complex patient*, *professional expertise and identity*, *system pressures* and *inflexible structures*. These themes were mapped to the SEM, demonstrating influences across individual and environmental levels. The second category ‘Adaptive Decision-Making Strategies’ included the themes *Mitigating risk* and *Relying on heuristics* capturing how HCPs navigate the uncertainty created by these influences. The theme, *Relational care*, operated between categories, illustrating how relationships both shape what decisions are possible (interpersonal level of SEM) and provide a means of navigating uncertainty.

**Conclusions:**

Clinical decision-making for people living with MLTC in emergency and acute settings is shaped by uncertainty arising from the interaction of factors operating across individual, interpersonal and environmental levels. To manage this uncertainty, staff employ a range of adaptive strategies. However, participants described this level of compensatory work as challenging and potentially insufficient on its own. Supporting decision-making and reducing reliance on adaptive responses may benefit from system‑level change, including improved community care, enhanced generalist expertise, opportunities for relational decision-making, better continuity and shared ownership of care, and flexible care pathways.

**Supplementary information:**

The online version contains supplementary material available at https://doi.org/10.1186/s12916-026-05236-6.

## Background

MLTC, also referred to as multimorbidity, is a growing issue [1–4] that creates challenges for health systems and those who work within them. At a health system level, MLTC are associated with increased hospital admissions [5, 6] longer hospital stays [7] and higher risks of treatment burden and polypharmacy [7–10] contributing to pressure on already stretched hospital services [11]. The definition of MLTC - the presence of two or more chronic conditions [12–14], does not reflect the heterogeneity that clinicians often encounter in practice. Previous international research across hospital, primary, and community-based care settings shows that clinical decision-making around MLTC is complex and shaped by physiological vulnerability [15, 16], symptom, treatment, and service interactions [17–20] and limited relevant guidelines [16, 21–24]. HCPs often struggle to reconcile single-condition recommendations with holistic patient needs, and existing pathways offer limited practical support due to their misalignment with the complexity of MLTC [17, 25–27]. This may result in fragmented coordination, poor continuity, limited multidisciplinary input [25, 28–30], and ultimately poorer clinical outcomes [31, 32]. Poor collaboration can compound these difficulties by limiting timely access to relevant patient information thereby constraining clinicians’ ability to make informed and timely decisions [17]. Furthermore, the need to involve multiple professionals, across different clinical specialties, may contribute to delayed discharge, problematic transitions and missed opportunities for integrated care [25, 26]. Together, these factors contribute to a clinical environment characterised by uncertainty. Prior healthcare research suggests that clinicians often have relatively low tolerance for uncertainty in such contexts and may respond by employing adaptive strategies which reduce uncertainty whilst allowing care to continue [33].

While existing literature has mapped the broader contextual and structural challenges of MLTC care, less attention has been paid to how decisions are made in real time at the point of hospital presentation. This qualitative study aimed to address that gap by examining how frontline hospital staff navigate clinical decision-making around referrals, admissions and care planning for people living with MLTC including what informs and constrains their choices and the approaches they use to manage complexity and uncertainty inherent in these decisions.

## Methods

This qualitative study used semi-structured interviews with HCPs to explore decision-making for people living with MLTC. The study was approved by the NHS Health Research Authority and Health and Care Research Wales (REC reference: 24/HRA/1483). This study is part of the ADMISSION collaborative, a programme funded by UK Research and Innovation and UK based National Institute for Health and Care Research (NIHR), aiming to improve care for people living with multiple long-term conditions. Based on five UK academic centres, ADMISSION brings together clinical, data, social, and computational sciences in partnership with public contributors with lived experience of MLTC [20].

### Selection and recruitment

We recruited 40 HCPs working within the UK National Health Service (NHS), the publicly funded healthcare system providing the majority of care across England, Scotland, Wales, and Northern Ireland. Participants were purposively sampled across professional roles, including physicians, nurses, allied health professionals, and managers. Participants worked in frontline settings such as emergency departments (hospital-based services providing immediate care for urgent and life-threatening conditions), acute assessment units (specialist units providing rapid assessment, diagnostic work-up, and short-term management for patients with acute medical needs), and related hospital services, involved in the rapid assessment and management of patients. The target sample size of up to 40 was not guided by a formal assessment of saturation (although later interviews tended to elaborate existing patterns rather than introduce substantively new topics) but to achieve diversity across professional roles, specialties, and geographical locations and volume and richness of qualitative data within the available time and resources.

Recruitment was undertaken through a combination of snowball sampling and network-based dissemination. It was initiated through the existing professional networks of the Principal Investigator (MW) and the ADMISSION team who emailed eligible contacts with details of the study and instructions to contact the lead researcher, SP, to express an interest in taking part. Recruited participants, in turn disseminated study information to colleagues within their own professional networks. Recruitment was subsequently expanded through the NIHR Research Delivery Network (https://www.nihr.ac.uk/support-and-services/support-for-delivering-research/research-delivery-network) and CHAIN network (https://www.chain-network.org.uk/). Study leaflets were also distributed at conferences attended by clinicians engaged in acute hospital care.

Eligible participants were English-speaking HCPs involved in secondary care decision-making for people living with MLTC within the NHS in England, Scotland, Ireland and Wales. Potential participants who expressed interest in the study were contacted with a project summary (Supplementary file 1) and a range of possible interview dates. Once a date was agreed, participants were emailed the participant information sheet (Supplementary file 2), which provided further details about the study, along with an informed consent form (Supplementary file 3) to complete and return if they were satisfied with the information provided prior to the interview.

The lead author (SP) and research team from the HealthTech Research Centre in Diagnostic and Technology Evaluation (HRC) (JS, NH, AS) had no prior relationships with any participants before study commencement. Participants were informed, at the start of the interview, that the HRC team comprised non-clinician researchers interested in understanding how clinicians make decisions when caring for people living with multiple long-term conditions, and that the study aimed to explore their experiences and perspectives.

### Data collection

Interviews were conducted online via Microsoft Teams between January and September 2024, lasting between 40 and 80 min. The lead interviewer (SP) joined from a home office, with a second interviewer from the HRC team also joining from their home or the HRC’s head office at Newcastle University. Participants were interviewed either at their workplace or at home, depending on their preference and availability. SP led the interviews with NH providing support including note-taking and clarifying questions. When NH was unavailable, AS or JS provided equivalent support. One interview (participant ID ADM_15) was led by NH and supported by AS. Detailed notes were taken during interviews by the supporting HRC methodologist, using a Rapid Assessment Sheet (RAP) as described in rapid qualitative research processes [34, 35]. With consent, interviews were video recorded. Participants were not reimbursed for their time.

### Interview guide

Interviews followed a semi-structured guide (Supplementary file 4) developed by members of the HRC team (SP, NH, JS). The guide was based on the HRC’s established approach to care pathway analysis [36], which explores current clinical practice, key decision points, and opportunities for pathway improvement. As MLTC does not correspond to a single diagnostic pathway, the interview schedule placed greater emphasis on decision-making processes, including factors influencing clinical judgement and how decisions are made for people living with MLTC attending hospital, while retaining flexibility to explore issues raised by participants. The schedule was reviewed and refined with input from the Principal Investigator (MW) and project team member SB, to ensure alignment with the study aims and relevance to acute care contexts. Two pilot interviews were conducted which resulted in no change to the interview schedule. Data from these two interviews are included in the analysis.

### Patient and public involvement and engagement (PPIE)

The study was developed with input from the HRC DTE Insight Panel comprising nine patients, carers, and members of the public, and the ADMISSION Patient Advisory Group (PAG), comprising fifteen people living with MLTC and informal carers. The Patient Advisory Group (PAG) has played a central role in shaping the ADMISSION programme from its inception through to delivery and dissemination. Their input led to important changes to the research design, including the addition of a qualitative workstream and a focus on hospital care pathways [20]. The HRC DTE Insight Panel reviewed the study protocol and participant materials, providing feedback on their clarity and accessibility. Although the study involved healthcare professionals, PPIE input helped ensure the study remained relevant and acceptable to people living with MLTC.

### Data processing and analysis

Recordings were automatically transcribed in Microsoft Teams and transcripts were imported into Otter.ai software (v3.53.0-240624), where they were pseudonymised and reviewed for accuracy. All transcripts were cross-checked in full against the audio by NH, AS, and SP through repeated reading and listening to identify and correct any transcription errors or omissions. The verified transcripts were then imported into NVivo (v14) for coding.

Consistent with the HRC’s translational approach to qualitative research, the study sought both to understand factors shaping clinical decision-making and care pathway choices and to explore how clinicians navigate the complexity of decision-making for people living with MLTC. Analysis followed Braun and Clarke’s six-phase reflexive thematic analysis approach [37]: (1) SP and NH independently read and coded an initial subset of three transcripts to support reflexive engagement with the data and explore and deepen interpretive perspectives. Following discussion of these early interpretations, SP undertook repeated reading and familiarisation with the remaining transcripts. (2) Coding then took place inductively and iteratively, with codes generated from participants’ accounts rather than a predefined framework, capturing both semantic and latent meanings. (3) Codes were developed into candidate themes through ongoing movement between the full dataset, coded extracts, and emerging patterns of meaning. (4) Themes were subsequently reviewed with ongoing input from NH and AS throughout the analytic process, including discussion of themes and critical reflection on interpretive development. (5) Themes were refined, defined, and named through discussion within the research team to ensure they were coherent, distinctive, and grounded in participants’ accounts. (6) Illustrative data extracts were selected and an analytic report produced based on an interpretive narrative that demonstrated how the themes addressed the research question. Consistent with reflexive thematic analysis, themes and interpretations were refined iteratively throughout manuscript development. An abductive approach was subsequently used to deepen interpretation through engagement with socioecological concepts. Themes relating to influences on and constraints to decision-making were mapped onto a simplified Social Ecological Model (SEM), collapsing Bronfenbrenner’s five ecological systems into individual, interpersonal, and environmental domains [38].

Findings were synthesised into an explanatory account of decision-making for people living with MLTC, from which a conceptual model was developed to illustrate how interacting influences shape decisions.

This study is reported in accordance with the COREQ (Consolidated Criteria for Reporting Qualitative Research) guidelines. The COREQ checklist is included as Supplementary file 5.

### Reflexivity statement

The research team comprised qualitative methodologists from the NIHR HealthTech Research Centre in Diagnostic and Technology Evaluation (HRC DTE), an academic geriatrician with expertise in MLTC and clinical trials involving older people, and a senior researcher with additional qualitative expertise from Newcastle’s AGE Research Group and NIHR Biomedical Research Centre. SP conducted the interviews and led the analysis, which was refined through ongoing discussion and critical reflection within the multidisciplinary team.

The paper’s lead author (SP) is a female researcher with a background (PhD) in cognitive neuroscience and a methodological role within an NIHR HealthTech Research Centre. Her disciplinary training has shaped a structured and analytical approach to understanding decision-making and her translational focus means she often prioritises practical, system-level insights over more theoretically expansive interpretations. However, her psychology background brings an awareness of the subjective nature of experience and the ways in which meaning is uniquely and contextually made by individuals. As a non-clinician, she occupied an outsider position in relation to participants. This critical distance meant she was initially less familiar with certain clinical contexts and assumptions, but it also enabled her to question taken-for-granted practices and attend closely to how clinicians articulated their reasoning. At the same time, this position may have limited her interpretation of some clinical nuances. Throughout the study, the author remained mindful of these influences by grounding interpretations in participants’ accounts and seeking clarification where needed.

## Results

### Description of sample

Forty HCPs, working at the initial point of hospital contact, participated in the study. Thirty-nine participants were practising clinicians, four of whom also held managerial responsibilities with one consultant managing acute medicine and ambulatory emergency care, one nurse manager in acute medicine, one occupational therapist managing emergency, acute and general medicine services, and one physiotherapist managing a frailty ward. One participant was a ward manager in an acute medicine department and had no clinical role.

A total of 18 individuals who expressed interest in participating and who were contacted with an invitation to take part, did not reply to the invitation and therefore did not participate in the study. Three separate individuals expressed interest after the data collection period had ended and therefore did not participate.

**Fig. 1:**
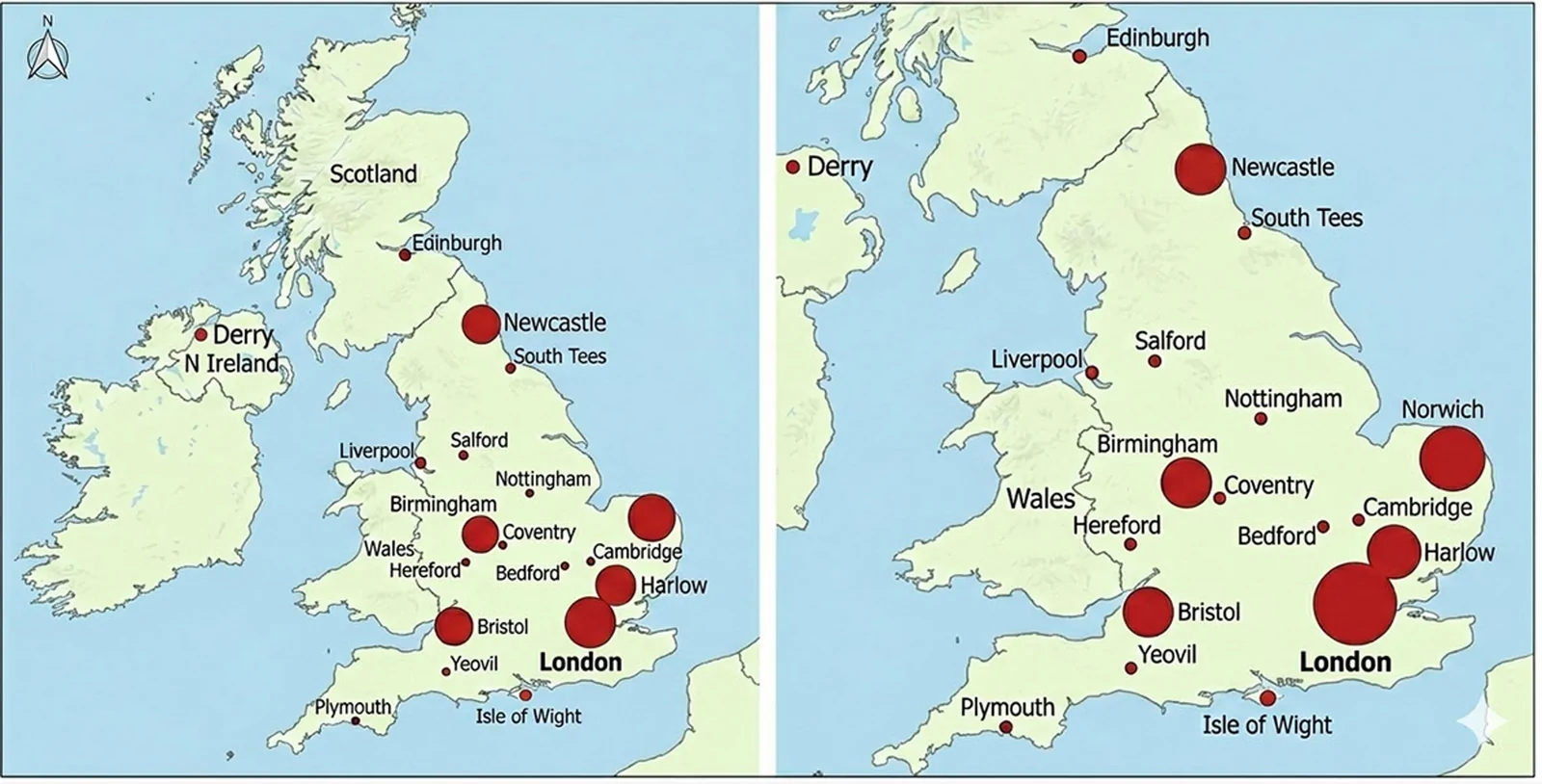
Map of participant locations within the UK (*n* = 40). Larger dots showing areas with higher numbers of participants. We acknowledge the use of Gemini (Google, https://gemini.google.com) to generate the visual map used for spatial representation of participant sites. Map created 12th May 2026

Participants represented a range of clinical departments (Table 1), roles (Table 2) and locations across England and the devolved nations (Fig. 1). Settings ranged between large urban teaching hospitals and smaller district general hospitals. Table 3 presents the professional, demographic and organisational characteristics of all participants. Participants had between 0.5 and 20 years of experience in their current role (median = 5.5 years), and 4 to 32 years in practice overall (median = 18 years).

**Table 1:** Clinical departments of study participants

Department	Description	(*n*)
Acute Medicine	Front-door acute medical assessment and short-stay care	15
Emergency Medicine	24-hour unscheduled and emergency care	13
Frailty	Specialist multidisciplinary services for older adults with frailty	4
Geriatric Medicine	Specialist inpatient and outpatient care for older adults	6
Admissions Unit	Rapid assessment/observation unit for new medical admissions	1
Cardiology	Specialist cardiovascular services	1
**Total**		**40**

**Table 2:** Professional roles of study participants

Role	Description	(*n*)
Advanced Clinical Practitioner	Senior non-medical clinician with advanced practice training (diagnosis, prescribing, autonomous decision-making)	8
Clinical Academic	Medical doctor with combined clinical and research roles	1
Consultant	Senior specialist physician with full independent practice	16
Senior Nursing Staff	Experienced nurses in leadership or advanced clinical roles	3
Postgraduate trainee doctor	Medical doctor undertaking postgraduate clinical training	7
Manager (non-clinical)	Service or operational managers within acute care	1
Pharmacist	Clinical pharmacists working in acute care	1
Allied Health Professionals	Includes physiotherapists, occupational therapists, and other regulated AHP roles	3
**Total**		**40**

**Table 3:** Professional, demographic and organisational characteristics of study participants

Participant ID	Role	Years in role	Years as clinician	Gender	Department	Location	Setting type	*Hospital size (no. beds)	^#^Urban/rural	*Hospital services and specialisms
ADM_Pilot_1	Consultant	10	27	Female	Respiratory and General internal Medicine	Birmingham	University Hospitals	1215	Urban	Type 1 major trauma centre. Specialities include orthopaedics, general surgery, colorectal, breast, upper gastroenterology, ear, nose and throat, maxillofacial, sarcoma, thoracic, burns and plastics, liver, renal, cardiac, neurosurgery and ophthalmology for adults. Regional centre for cancer, trauma, renal dialysis, burns and plastics and solid organ transplantation.
ADM_Pilot_2	Consultant	6	19	Female	Geriatric Medicine	Newcastle	DGH	580	Urban	Services include medical, surgical, maternity and gynaecology, services for children and young people, end of life and critical care.
ADM_1	Consultant	13	26	Male	Acute Medicine	Birmingham	University Hospitals	1215	Urban	Type 1 major trauma centre. Specialities include orthopaedics, general surgery, colorectal, breast, upper gastroenterology, ear, nose and throat, maxillofacial, sarcoma, thoracic, burns and plastics, liver, renal, cardiac, neurosurgery and ophthalmology for adults. Regional centre for cancer, trauma, renal dialysis, burns and plastics and solid organ transplantation.
ADM_2	Consultant	14	30	Male	Acute Medicine	Birmingham	DG and teaching hospital	550	Urban	Acute and general medicine, cardiology, elderly care, stroke, paediatrics, respiratory medicine, diabetes, obstetrics, gynaecology, maternity, ophthalmology, orthopaedic surgery, colorectal and upper GI surgery, urology and vascular surgery.
ADM_3	Consultant	16	25	Female	Respiratory Medicine	Liverpool	Teaching hospital	> 750	Urban	Urgent, emergency, major trauma. Specialist services include nephrology, renal transplantation, nuclear medicine, haematology, lithotripsy, dermatology, urology. National centre for ocular oncology, pancreatic, urological, testicular, anal and oesophago-gastric cancers, specialist palliative care, specialist radiology and specialist pathology and chemotherapy services.
ADM_4	Consultant	15	30	Female	Acute Medicine and Infectious Diseases	Nottingham	University Hospital	975	Urban	Specialist services including stroke, renal, neurosurgery, cancer services, maternity and trauma.
ADM_5	Consultant	8	20	Female	Acute Medicine	Hereford	DGH	240	Urban/rural	Acute and emergency medicine. Specialist services including older people’s care, services for children and young people, end of life care, maternity, surgery.
ADM_6	Consultant	12	17	Female	Geriatric Medicine	Salford	University Hospital	90	Urban	Trauma and essential recovery services including physiotherapy, occupational therapy, psychological support and nutrition and hydration assistance.
ADM_7	Trainee Advanced Clinical Practitioner (ACP)	1	13	Female	Frailty (Hospital at Home)	South Tees	DG and teaching hospital	Saves around 1000 beds per month	Urban/rural	Frailty, complex and acute illness.
ADM_8	Postgraduate trainee doctor	3	15	Female	Acute Medicine	N. Ireland	Teaching hospital	508	Urban/rural	Acute, emergency and specialist services including medical imaging, surgery, maternity, oncology, physiotherapy.
ADM_9	Consultant	10	22	Female	Geriatric Medicine	Cambridge	University Teaching hospital	1165	Urban/rural	Major trauma centre, specialises in rare and complex conditions, organ transplants, cancer care, and neurosciences.
ADM_10	Consultant	1.5	17	Female	Acute Medicine	Newcastle	Teaching and tertiary referral hospital	673	Urban	Emergency medicine and trauma. Specialisms include maternity, paediatrics, neurosciences, women’s health. Further services include breast screening, gynaecology, ophthalmology, burns unit.
ADM_11	Postgraduate trainee doctor	9	25	Female	Emergency Medicine	Bedford	DGH	400	Urban/rural	Services include emergency medicine, acute medicine, maternity, paediatrics and surgery.
ADM_12	Consultant	6	22	Female	Acute Medicine and Intensive Care	London	DG and teaching hospital	320	Urban	Emergency medicine. Departments include care of older people, stroke rehabilitation, maternity, oncology, haematology, gastroenterology, surgery, gynaecology, medical assessment.
ADM_13	Consultant and manager	6	18	Female	Acute Medicine & Ambulatory Emergency care	London	DG and teaching hospital	320	Urban	Emergency. Departments include care of older people, stroke rehabilitation, maternity, oncology, haematology, gastroenterology, surgery, gynaecology, medical assessment.
ADM_14	ACP	6	13	Male	Emergency Medicine	London	DG and teaching hospital	320	Urban	Emergency. Departments include care of older people, stroke rehabilitation, maternity, oncology, haematology, gastroenterology, surgery, gynaecology, medical assessment.
ADM_15	Consultant	6	19	Female	Geriatric and Acute Medicine	London	DG and teaching hospital	320	Urban	Emergency. Departments include care of older people, stroke rehabilitation, maternity, oncology, haematology, gastroenterology, surgery, gynaecology, medical assessment.
ADM_16	Nurse manager	20	32	Female	Acute Medicine	London	DG and teaching hospital	320	Urban	Emergency. Departments include care of older people, stroke rehabilitation, maternity, oncology, haematology, gastroenterology, surgery, gynaecology, medical assessment.
ADM_17	Occupational therapist and manager	11	15	Female	Emergency, Acute and General Medicine	Edinburgh	Teaching hospital	570	Urban	Services include acute medicine, breast surgery, cardiology, colorectal surgery, diabetes, dietetics, endocrinology, endoscopy, gastroenterology, general medicine, general surgery, haematology, infectious disease, laboratories, medicine of the elderly, oncology, palliative. medicine, renal dialysis, respiratory medicine, rheumatology, stroke medicine, urology.
ADM_18	Nurse consultant	1.5	15	Female	Geriatric Medicine	Coventry	University Hospitals	917	Urban/rural	Emergency and elective care. Specialise in cardiology, neurosurgery, stroke, joint replacements and in vitro fertilisation.
ADM_19	ACP	3.5	10	Male	Emergency Medicine	London	DG and teaching hospital	320	Urban	Emergency medicine. Departments include care of older people, stroke rehabilitation, maternity, oncology, haematology, gastroenterology, surgery, gynaecology, medical assessment.
ADM_20	ACP	0.5	25	Female	Acute Frailty	Isle of Wight	DGH	266	Urban/rural	Services include emergency department, urgent treatment centre, surgery, intensive care, comprehensive maternity, special care baby unit, paediatric services.
ADM_21	Advanced Pharmacist	5	25	Female	Frailty	Harlow	DGH	418	Urban/rural	Services include child health, medicine for the elderly, general medicine, cardiology and coronary care, general surgery, urology, trauma and orthopaedics, plastic surgery, obstetrics and gynaecology, ophthalmology, ENT, maxillo-facial surgery, orthodontics, intensive and high dependency care, accident and emergency, radiology, diagnostics.
ADM_22	Clinical Research Fellow	2	15	Female	Emergency Medicine	London	University hospital	900 (Trust)	Urban	Large number of services across two sites including general and acute, maternity, older people’s care, services for children and young people, end of life care, diagnostic imaging and critical care.
ADM_23	Advanced Nurse Practitioner	4	12	Female	Emergency Medicine	London	DG and teaching hospital	320	Urban	Emergency. Departments include care of older people, stroke rehabilitation, maternity, oncology, haematology, gastroenterology, surgery, gynaecology, medical assessment.
ADM_24	Physiotherapist and manager	9	14	Female	Frailty	Harlow	DGH	418	Urban/rural	Services include child health, medicine for the elderly, general medicine, cardiology and coronary care, general surgery, urology, trauma and orthopaedics, plastic surgery, obstetrics and gynaecology, ophthalmology, ENT, maxillo-facial surgery, orthodontics, intensive and high dependency care, accident and emergency, radiology, diagnostics.
ADM_25	ACP	2	30	Female	Geriatric Medicine	Harlow	DGH	418	Urban/rural	Services include child health, medicine for the elderly, general medicine, cardiology and coronary care, general surgery, urology, trauma and orthopaedics, plastic surgery, obstetrics and gynaecology, ophthalmology, ENT, maxillo-facial surgery, orthodontics, intensive and high dependency care, accident and emergency, radiology, diagnostics.
ADM_26	ACP	1	8	Male	Acute Medicine	Yeovil	DGH	345	Urban/rural	Services include general medicine, cardiology, general surgery, orthopaedic surgery, trauma and paediatrics, with an emphasis on enhanced recovery.
ADM_27	ACP	1	8	Female	Emergency Medicine	Norwich	University hospital	1200	Urban/rural	Full range of acute clinical services, including more specialist services such as oncology and radiotherapy (including brachytherapy), neonatology, trauma and orthopaedics, plastic surgery, vascular surgery, robotic surgery, bone marrow transplants, interventional. radiology, specialist cardiology, paediatric medicine and surgery
ADM_28	Physiotherapist	0.5	4	Female	Emergency Medicine	Harlow	District General hospital	418	Urban/rural	Services include child health, medicine for the elderly, general medicine, cardiology and coronary care, general surgery, urology, trauma and orthopaedics, plastic surgery, obstetrics and gynaecology, ophthalmology, ENT, maxillo-facial surgery, orthodontics, intensive and high dependency care, accident and emergency, radiology, diagnostics.
ADM_29	Postgraduate trainee doctor	5	Registrar	Female	Rotation (recently, Frailty and Cardiology)	Newcastle	Teaching hospital	800	Urban	Services range from joint replacement to heart and chest surgery, innovative cancer care and multi-organ transplantation. Specialists also offer innovative treatments for complex liver, pancreatic, kidney, lung and heart diseases.
ADM_30	Postgraduate trainee doctor	8	Registrar	Female	Emergency Medicine	Plymouth	Teaching hospital	1000	Urban/rural	Major trauma centre. Services include emergency, urgent care, surgery, maternity, diagnostics.
ADM_31	Consultant	0.5	17	Male	Emergency Medicine	Norwich	University hospital	1200	Urban/rural	Full range of acute clinical services, including oncology and radiotherapy (including brachytherapy), neonatology, trauma and orthopaedics, plastic surgery, vascular surgery, robotic surgery, bone marrow transplants, interventional radiology, specialist cardiology. paediatric medicine and surgery
ADM_32	Postgraduate trainee doctor	1	7	Male	Emergency Medicine	Norwich	University hospital	1200	Urban/rural	Full range of acute clinical services, including oncology and radiotherapy (including brachytherapy), neonatology, trauma and orthopaedics, plastic surgery, vascular surgery, robotic surgery, bone marrow transplants, interventional radiology, specialist cardiology. paediatric medicine and surgery
ADM_33	Consultant	0.5	19	Female	Medicine Admissions	Bristol	Teaching hospital	581	Urban	General and acute medicine and surgery, specialist surgery, critical care, trauma and orthopaedics and accident and emergency services. Centre for cardiothoracic services.
ADM_34	Consultant	0.5	9	Female	Acute Medicine	Bristol	Teaching hospital	581	Urban	General and acute medicine and surgery, specialist surgery, critical care, trauma and orthopaedics and accident and emergency services. Centre for cardiothoracic services.
ADM_35	ACP	6	21	Female	Emergency Medicine	Norwich	University hospital	1200	Urban/rural	Full range of acute clinical services, including more specialist services such as oncology and radiotherapy (including brachytherapy), neonatology, trauma and orthopaedics, plastic surgery, vascular surgery, robotic surgery, bone marrow transplants, interventional radiology, specialist cardiology, paediatric medicine and surgery.
ADM_36	Ward Manager	2	6	Male	Acute Medicine	Bristol	Teaching hospital	581	Urban	General and acute medicine and surgery, specialist surgery, critical care, trauma and orthopaedics and accident and emergency services. Centre for cardiothoracic services.
ADM_37	Postgraduate trainee doctor	3	25	Male	Emergency Medicine	Norwich	University hospital	1200	Urban/rural	Full range of acute clinical services, including more specialist services such as oncology and radiotherapy (including brachytherapy), neonatology, trauma and orthopaedics, plastic surgery, vascular surgery, robotic surgery, bone marrow transplants, interventional radiology, specialist cardiology, paediatric medicine and surgery.
ADM_38	Postgraduate trainee doctor	6	Registrar	Male	Emergency Medicine	London	University hospital	939	Urban	Centre of excellence for neuro, stroke, and maternity. Also provide elective and emergency care. Broad range of other services.

* Where participants did not provide this information, it was obtained from the relevant hospital Trust website or Care Quality Commission (CQC) inspection report.^#^ Where participants did not provide this information, it was obtained from Office for National Statistics (ONS) 2021 Rural Urban Classification for England

### Themes

Seven themes were constructed and organised into two overarching categories. The first, ‘Factors Influencing Decision-Making’, captures the factors participants described as shaping clinical judgements and care pathway decisions. The second, ‘Adaptive Decision-Making Strategies’, focuses on how participants responded to and negotiated the uncertainty arising from the interaction of these factors. The Factors influencing decision-making category included the themes ‘the complex patient’, ‘professional experience and identity’, ‘system pressures’, and ‘inflexible structures’. Analysis at this level was primarily descriptive but drew attention to aspects of decision-making that were consistently represented as fundamental to clinical practice. The adaptive strategies category comprised ‘mitigating risk’ and ‘relying on heuristics’. Analysis at the adaptive-strategy level was more interpretive, synthesising clinicians’ descriptions of how they actively respond to uncertainty and system pressures into patterns of strategic adaptation. The theme, ‘relational care’, cut across both categories, functioning both as a factor influencing decision-making and as an adaptive strategy clinicians drew on in practice.

In this paper, where the term “single-condition” is used, this refers to clinical guidelines and pathways organised around individual conditions, while the term “single-organ” refers to hospital specialisms organised around organ-based disciplines such as cardiology or neurology.

#### Category 1: Factors influencing decision making

Factors influencing decision-making operated across multiple levels of the social ecological model (SEM), shaping practice in interconnected ways. We therefore abductively mapped these factors onto the SEM framework, organising them across the individual, interpersonal, and environmental levels to provide a coherent structure for interpretation.

#### Individual level

##### The complex patient

This theme captures how the clinical complexity of people living with MLTC fundamentally complicates decision-making. Participants described many MLTC presentations as ‘atypical’ where patients do not fit neatly into diagnostic categories or single-condition pathways, it is often unclear how different conditions are contributing to symptoms, and clinicians struggle to determine which issue requires the most urgent attention.

“Symptoms [can be] really nonspecific. And so often, when a patient initially presents if you have limited information, it’s really difficult to be sure of if it is a single entity that’s driving that, and what could be contributory from other organs or diseases that the person might also be suffering with”. – ADM_Pilot_1 (consultant_acute_medicine).

Risk and uncertainty emerged as central features of clinicians’ accounts of patient complexity. These were shaped by multiple factors, including the interactions between coexisting conditions and their treatments, and differences in what is considered ‘normal’ or ‘baseline’ for individuals without MLTC compared with those with MLTC. This can substantially alter what is considered clinically appropriate, such that referrals that would ordinarily be indicated are no longer pursued.

“If that same patient wouldn’t be a candidate for a pacemaker, because they had advanced malignancy… then I wouldn’t make that referral to cardiology” – ADM_6 (consultant_geriatric_medicine).

Clinicians described having to make difficult trade-offs, such as deciding which condition to prioritise and balancing the acute, immediate needs of the patient with the wider, ongoing management of MLTC. Narratives around this conveyed a sense of uneasy compromise, as clinicians described juggling competing demands.

“So they have multiple different conditions, but one of those conditions is their lungs and their respiratory problems. You’re probably focusing more on that, and again, kind of thinking about the other things in the background, but acknowledging that there’s one particular problem at that time” – ADM_29 (postgraduate_trainee doctor_frailty).

##### Professional experience and identity

Uncertainty and discomfort in decision-making for people living with MLTC was not experienced uniformly. Seniority, confidence, and clinical expertise shaped how participants navigated complexity. Staff holding more senior positions described feeling more able to take ownership of complex presentations and make decisions in situations characterised by uncertainty. As one acute consultant explained “There’s some things I’m more comfortable with [than more junior colleagues]… I’ll manage things like heart failure, pneumonias, chronic kidney disease, frailty, cancers… I’ll do some simple tests to understand the problem better.” – ADM_1 (consultant_acute_medicine).

More junior colleagues described having less autonomy and greater anxiety about undertaking decisions that involved risk. However, narratives did not suggest that senior clinicians experience *less* uncertainty than their junior counterparts. Rather, they recognise complexity and uncertainty as inherent to decision-making for people living with MLTC and are more at ease with tolerating this uncertainty and acting within it.

Job role and clinical setting also shaped how participants approached decision-making. Emergency and acute physicians described a necessary focus on the immediate, acute problem, often setting aside wider MLTC considerations. For many, this was framed not as a personal preference but as a requirement of the role. As one acute medicine consultant explained:

“As an acute medic, my key skill I find is to unpick what’s wrong with them today, not understand what’s happened to them in their entirety.” – ADM_1 (consultant_acute_medicine).

In contrast, participants working in geriatric or frailty services described a much greater comfort with complexity and a professional identity grounded in holistic, whole-person care. They emphasised that managing MLTC is embedded in their training and everyday practice:

“The other people in their registrar years learn how to do endoscopies and angiograms, we spend five years learning how to manage medical complexity, how to manage a multidisciplinary team, how to deliver a comprehensive geriatric assessment using the skill of the MDT to develop a personalised care plan for patients… a lot of transferable domains that apply to people with multiple long-term conditions quite naturally” – ADM_9 (consultant_geriatric_medicine).

#### Interpersonal level

##### Relational care

Existing relationships were highly influential in shaping decision-making. Participants spoke about the importance of knowing who to contact to support a person living with MLTC, both within their own team and in other departments. One of the main factors influencing care-pathway decisions was having a clinician or service willing to assume ownership. However, participants noted that this was often difficult to secure for people living with MLTC, who sit across multiple specialties. Clinicians also described reluctance among teams to take on complex patients, which could complicate decision-making and create additional work and time pressure for the clinician trying to coordinate care:

“I think probably getting the specialty to accept the patient can be quite difficult. If they’ve got multiple things, then they might say, ‘Oh no, they’re under gastro,’ or ‘No, they’re under the surgeons.’ And then you’re spending all day battling between who feels they should take responsibility for the patient.” – ADM_27 (ACP_emergency).

Relational care also extended to patients and their families, and participants emphasised the importance of treating patients as active partners in decision-making, with patient priorities emerging as one of the main influences on clinical decisions.

“The main approach would be looking at… what their priorities for their life are so what they want us to treat, what they don’t want us to look into, and tailoring our approach around their preferences.” – ADM_25 (ACP_geriatric_medicine).

#### Environmental level

Factors influencing decision-making within the environmental domain of the decision-making ecology operated at both organisational and wider health-system levels.

##### System pressures

Barriers to effective clinical decision-making for people living with MLTC were often tied to limited hospital capacity, staffing shortages, and gaps in essential services. These pressures are felt most acutely in emergency departments, where the complex needs of MLTC patients require more time and flexibility, clashing with the fast-paced, high-turnover environment.

“When you got multiple [long term conditions], you don’t know where to start, and the [emergency] department is busy… I don’t have the bandwidth… to deal with all your 25 complaints… with an exploding department.” – ADM_19 (ACP_emergency_medicine).

Time-based performance targets, like the NHS four-hour rule in accident and emergency departments and the 72-hour rule in acute medicine exacerbate the pressure, where hospital staff must make complex decisions for people living with MLTC within severely constrained timeframes. As one senior emergency nurse explained:

“We’ve got this four-hour target in A&E… within the first hour we… do the examination …, investigations, and start early referrals to… the right specialty. By two hours, that should be done, and by three hours someone… [asks], ‘Do you have a plan for this patient?’. But with people who have multiple conditions, it can be quite tricky.” – ADM_23 (senior_nurse_emergency_medicine).

Participants described a sense of ‘blockage’ in the health system that constrains options, especially for people living with MLTC. These blockages stem not only from in-hospital service limitations and inflexible or inadequate care pathways, but also from insufficient social and community care provision including limited access to preventative care, a shortage of hospices and care home or community services. In this context, HCPs may be hesitant to discharge complex patients without confidence that adequate safety nets are in place, leading to longer hospital stays and further congestion in discharge pathways.

“There’s a bottleneck at discharge… not for the simple person who had an appendix out now is going home, who’s 30 years old. It’s the people who have got uncontrolled diabetes that now need to be on insulin, but they don’t have the dexterity to do it themselves - they need a district nurse.” – ADM_38 (postgraduate_trainee doctor_emergency_medicine).

##### Inflexible structures

Participants described making decisions in an environment where the structure of hospital services is fundamentally misaligned with the needs of people living with MLTC. Many participants described a healthcare system organised around single-organ specialisms. This was seen to foster fragmented care, relying heavily on referrals and reinforcing the separation, or siloing, of different parts of the health system.

“We no longer believe that general medicine exists. Please tell from the sarcasm in my voice that this is rubbish. But the hospital no longer believes that general medicine exists, and everyone has to belong to a speciality.” – ADM_4 (consultant_acute_medicine).

Participants described how the health system’s single-organ focus makes it harder to address how multiple conditions interact or how treatment plans for one disease may conflict with another. This challenge is compounded by the persistence of single-condition guidelines, which offer little direction for managing care in people with multiple conditions. One participant described healthcare decisions as “piecemeal,” with each specialist focusing only on their own area and no-one taking an overarching view of the patient.

However, where general, geriatric and frailty teams were involved, participants saw a more ‘joined-up’ approach, both in how HCPs make clinical decisions for people living with MLTC and in how the healthcare system itself is structured. Participants from these settings described a philosophy of care which is marked by clearer, more realistic care planning and in which care is more continuous and person-centered.

“We look a bit more holistically at the patient, because [they may] have a social issue as well… it’s looking at the whole package of the patient, rather than necessarily what might be the presenting complaint.” – ADM_21 (pharmacist_frailty).

#### Summary of factors influencing decision-making

These themes describe how HCPs must make complex decisions for people living with MLTC who require greater time, attention, and resources in a system that is under-resourced and organised around single-condition pathways. These factors interact to produce a decision-making ecosystem which is characterised by profound uncertainty. Some participants conveyed a tension between moral and professional obligations to deliver optimal patient care, and the practical limitations on their ability to do so. Caught between internal motivations to “do the right thing” and external systemic pressures, with people living with MLTC at the centre of this tension, participants described relying on a range of adaptive strategies to support their decision-making.

#### Category 2: Adaptive decision-making strategies

##### Relying on heuristics

In the context of uncertainty created by multiple long-term conditions (MLTC), and the expectation that this complexity is managed within a limited time, participants described relying on heuristics - rules, mantras, protocols, and plans, to guide decision-making. A key heuristic was defining decisions through role boundaries, often expressed as “this is what we do”. In emergency and acute care, this typically meant focusing on the immediate problem or the organ system causing concern, rather than the wider set of conditions thus narrowing the scope of care to something more manageable. Structured approaches such as rapid assessment fast treatment (RAFT) and airways, breathing, circulation and disability (ABCD), directed attention towards immediate risks and next steps, enabling decisions to be made more quickly and defining the limits of care and responsibility.

For HCPs working within geriatric and frailty services, the comprehensive geriatric assessment acted as a “rule book” that allowed clinicians to step outside the usual time pressures and take a broader view of the patient. This was described by an advanced frailty pharmacist as “stopping the [emergency department] clock” (ADM_21), creating space to consider social, functional, psychological, and patient-prioritised factors alongside clinical needs.

Tools such as advanced care or escalation plans also provided a clearer pathway through uncertainty, helping clinicians align decisions with patient context and goals.

“so if they have… that kind of ReSPECT chart… [it can] be very, very helpful, because then we know that this is what we’re dealing with. This is how we need to approach this patient.” – ADM_11 (postgraduate_trainee_doctor_emergency_medicine).

##### Mitigating risk

In response to the underlying tension between the complexity of MLTC and a healthcare system organised around speed and efficiency, participants described a process of risk mitigation, often characterised as “covering all the bases” with the aim of reducing uncertainty by doing more rather than less. This included ordering multiple investigations, admitting patients “just in case,” and involving multiple specialties early. As one postgraduate trainee emergency doctor summarised, “So you’ve got loads of comorbidities and don’t really know what [the] problem is. So therefore we do investigations.” – ADM_38. This was referred to, by one participant, as a ‘scattergun’ approach.

MLTCs were understood to increase the risk of deterioration and the cumulative effect of comorbidities meant that patients were seen as more vulnerable and closer to “tipping over,” reinforcing a tendency to prioritise avoiding missed diagnoses over avoiding unnecessary admission.

“We’re so busy trying to cover our own backs and go, ‘What if we’ve missed something? what if there’s something there that needs to happen?’.” – ADM_4 (consultant_acute_medicine).

Where alternatives such as same day emergency care, geriatric input, or virtual wards were accessible, participants were more likely to discharge, using these services as a form of safety netting.

However, this risk-mitigation approach was described as time-consuming, with repeated investigations and iterative decision-making prolonging care pathways for both HCPs and patients:

“If you take the approach of doing an investigation, it being done, waiting for the results, coming back thinking about it and going, ‘Oh, it’s not that,’ and then sending out another referral for another investigation, it’s a very, very prolonged journey for that patient.” - ADM_Pilot_1 (consultant_acute_medicine).

##### Relational care

In a system not designed for MLTC, reliance on others was not considered optional but as necessary. As one postgraduate trainee doctor in acute medicine reflected, “no single person can do all these jobs for your patients… all the system has to be involved in managing a patient with multiple comorbidities” – ADM_8. Relationships with colleagues within and across teams, as well as with community services, patients, and families, became key to navigating care. These relationships helped clinicians access information, seek advice, share responsibility, and make decisions in situations where uncertainty was high and time was limited. Multidisciplinary team (MDT) working was described as an ideal form of relational care, drawing together varied clinical perspectives to enable coordinated, context-aware care across physical and mental health, medication safety, social factors, and prognosis.

Strong, established relationships, particularly with trusted “go-to” individuals, were described as especially valuable. These were individuals that participants felt they could approach informally for rapid advice, clarification, or support when managing complex cases. These relationships provided a reliable source of clinical judgement, often enabling quicker, more coordinated decision-making than formal pathways alone:

“So my usual approach actually is, yes, to have an understanding of what the guidelines would say, but probably actually go and speak to one of my colleagues in the organs of note that I was interested in and ask whether they thought it would be a reasonable referral… So, you know, what you want is to identify colleagues that are like minded and want to unpick this with you.” – ADM_1 (consultant_acute_medicine).

Patients and families themselves were described as key contributors to decision-making with their knowledge, preferences, and lived experience helping to reduce uncertainty and shape care:

“They can tell you actually, I’ve tried this drug before, it doesn’t work.” – ADM_12 (consultant_acute_medicine).

A further aspect of relational care was the practice of “passing the baton” - transferring responsibility to others when limits were reached. This could involve referring to specialists, other teams, or services perceived as better equipped to manage complexity such as general or geriatric medicine, frailty or same day emergency care (SDEC) providing reassurance that it would be dealt with and relieving the individual burden of uncertainty:

“Patients coming with breathlessness or chest pain go to SDEC… If I put them on that list for a SDEC, I can sleep well.” – ADM_3 (consultant_acute_medicine).

At the same time, relational care for people living with MLTC was not without cost. Participants described the labour involved in coordinating care, contacting multiple teams, negotiating input, and navigating availability, particularly in busy environments or when services were closed. This could delay decision-making and contribute to longer hospital stays:

“I think the difficulty with frailty, with a lot of comorbidities, is when we use quite a lot of transaction and negotiation, it delays the whole decision making, and then they stay here longer then they [become] deconditioned.” – ADM_24 (physio_manager_frailty).

### Conceptual model

**Fig. 2:**
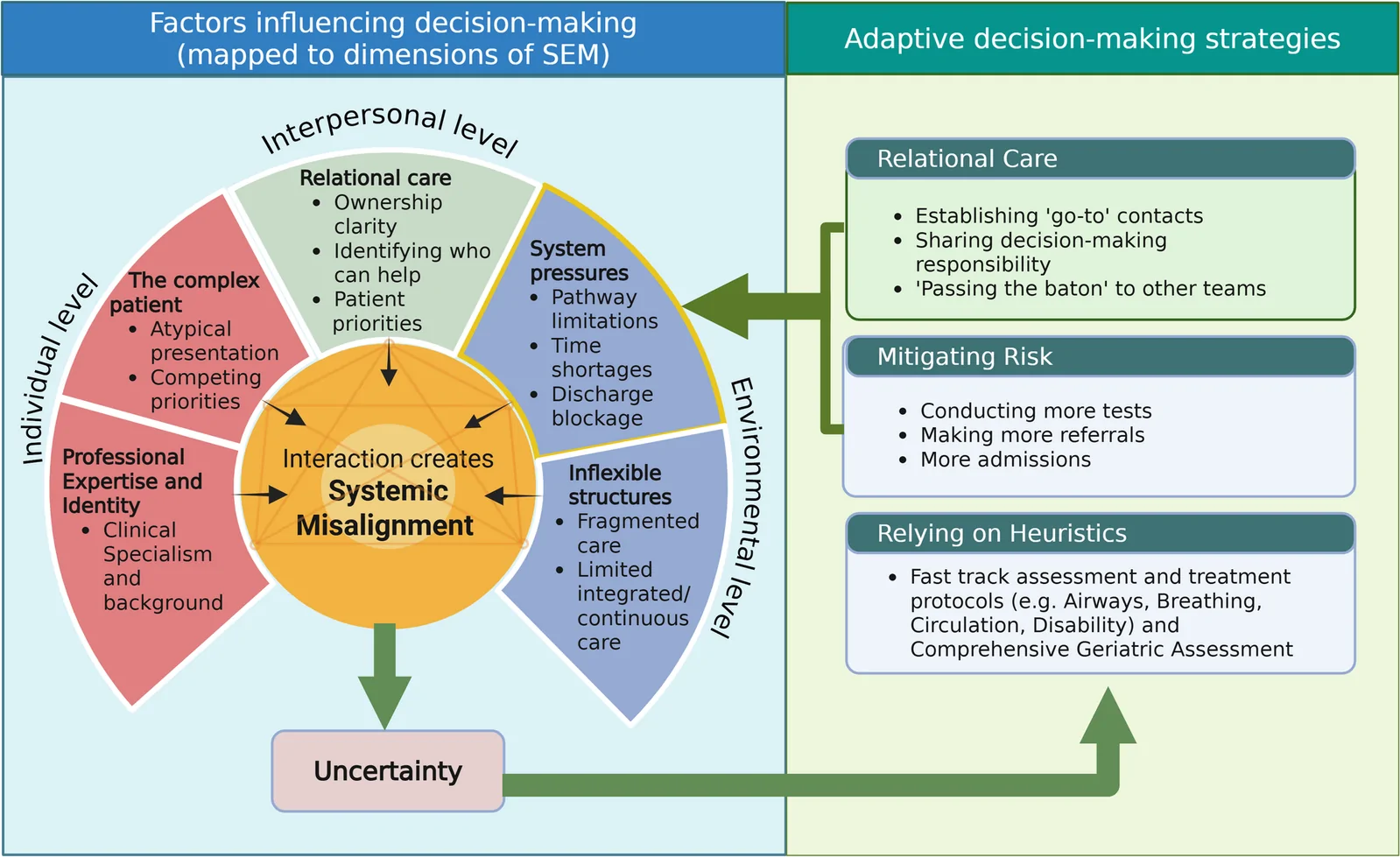
Conceptual model showing how factors influencing decision-making, mapped to SEM domains, work and interact to create uncertainty, and how clinicians respond through adaptive decision making strategies. The time and resource demands involved in building and maintaining relationships, alongside the use of risk-averse strategies, contribute to and reinforce existing system pressures. Created in https://BioRender.com

Participants’ accounts of decision-making around referrals, admissions, and care planning for people living with MLTC depict a dynamic system shaped by overlapping and interacting influencing factors and decision-making approaches. These relationships are illustrated in Fig. 2, where the themes within the category ‘Factors influencing decision-making’ are mapped onto the domains of the SEM.

Although we have presented factors influencing decision-making across the domains of the SEM, in reality the boundaries are blurred: factors cut across levels and compound one another. The misalignment between the needs of people living with MLTC and the fragmented structure of hospital services illustrates the interaction between individual-level complexity and organisational-level structures within the socioecological model. Furthermore, the complexity of MLTC means that patients often require more investigations, more treatments, the involvement of more teams, and more time and resources. These demands intensify existing system pressures and require relational care approaches. Fragmented care structures mean that this work cannot be delivered continuously or coherently. In this context, HCPs frequently depend on existing informal relationships and professional networks to coordinate care. Multi-specialist input and multiple appointments add time and pressure at both the individual and organisational level. This illustrates how patient complexity, interpersonal relationships, and organisational structures interact, with challenges arising not from any single factor but from their combined effects. This interplay produces a culture of uncertainty in which HCPs must make decisions without a coherent pathway to guide them. In response, clinicians draw on a set of compensatory mechanisms, risk-averse decisions, heuristics, and relational work. These are not discrete strategies but interconnected ways of navigating uncertainty and distributing responsibility. For example, ordering multiple tests or admitting a patient “just in case” is often closely tied to relational work: involving other teams, seeking specialist input, or using MDT structures to ensure that all aspects of a patient’s needs are considered.

While heuristics act as cognitive shortcuts that support rapid decision-making and limit the need for extensive input, risk-averse practices tend to increase referrals and admissions. Substantial work is also invested in building and sustaining relationships, negotiating ownership, and enabling collaborative decision-making across teams. Much of this “invisible work” falls outside clinicians’ formal roles and contributes to wider system pressures.

## Discussion

This study explored how healthcare professionals in emergency and acute care settings of UK hospitals make decisions around people living with MLTC who attend hospital and the factors which influence these decisions. Using the SEM, we highlight that clinical decision-making is shaped by interacting individual, interpersonal and environmental factors with clinicians drawing on relational and adaptive strategies to navigate complexity and uncertainty. A key insight from this work is the systemic misalignment between hospital systems and processes and the needs of people living with MLTC, with clinicians having to invest substantial time and energy in adaptive responses to circumvent this misalignment.

Decision-making for people living with MLTC was experienced as complex and non-linear, shaped by atypical clinical presentations, competing clinical priorities, fragmented pathways, and limited community and social care capacity. Clinicians described navigating systemic misalignment through compensatory strategies such as risk-averse decision-making, discipline-specific heuristics, and collaborative negotiation. While these strategies enabled decisions to progress in uncertain situations, participants suggested they may contribute to ongoing system pressures and fragmentation.

### Findings in relation to previous research

Our findings are consistent with those from previous research showing that clinical decision-making for people living with MLTC is shaped by complex and interacting clinical presentations and physiological vulnerability, alongside unclear care pathways and lack of clear guidelines in MLTC decision-making [15, 16, 21–24]. Ethical and practical tensions around the need to balance competing demands, including those between acute and chronic needs, and ideal care versus organisational constraints have been noted previously in MLTC care [22]. Similarly our findings around the constraints of limited resources and systemic pressures align with previous research, which has highlighted how workforce shortages, time pressures, and fragmented funding structures restrict the delivery of coordinated, patient-centred care for people living with MLTC [17, 22, 26, 39]. Consistent with previous research, MLTC was reported to create complex and fragmented care pathways that strain standard service models and health system resources, complicating clinical decision-making and limiting available options [17, 23, 28]. Whilst the finding that system constraints and structural inflexibility characterise the health system and complicate optimal patient care is not novel, and anecdotal accounts of this pervade the media, our study does not seek to introduce new concepts. Rather, it reinforces how limiting these factors are for HCPs making decisions about complex patients and adds depth through the presentation of clinicians’ perspectives in their own words and the use of illustrative quotations. The salience of these challenges for HCPs working in frontline clinical practice is evident in participants’ narratives. Rather than reiterating the factors influencing decision-making as discrete barriers, our study shows how these influences interact across levels of the healthcare system to generate pervasive uncertainty, which in turn shapes how clinical decisions are made in real time. By mapping these influences to the SEM, we demonstrate that uncertainty is not attributable to any single factor, but emerges from their dynamic interplay. This uncertainty is consequential as it drives reliance on adaptive strategies. Our conceptual model illustrates that these strategies may not simply be responses to system pressures but could also contribute to sustaining them through ongoing cycles of adaptation.

Previous research has suggested that clinicians with lower tolerance for ambiguity may employ defensive cognitive strategies and favour structured or rational decision-making approaches over more intuitive ones [33, 40], and that relationship-based approaches are particularly important in healthcare systems characterised by high levels of uncertainty [41]. However, these studies have not shown how clinicians draw simultaneously on heuristics, risk-management practices, and relational resources to navigate this uncertainty in everyday practice.

Our findings resonate with work on organisational resilience, particularly Braithwaite and colleagues’ conceptualisation of healthcare systems as fundamentally adaptive and reliant on the capacity of frontline staff to anticipate, respond, and adjust to challenging conditions [42–44]. Participants described continually modifying practices in response to time pressure, resource limitations, fragmented systems, and pathway inadequacies to maintain care delivery. This aligns with distinctions within resilience engineering between “work as imagined” (WAI) - the formal procedures, pathways, and guidelines envisioned by policymakers and organisations - and “work as done” (WAD), referring to the situated and often improvised reality of frontline practice [45]. Our findings illustrate this distinction clearly. Participants frequently described clinical guidelines and standardised pathways as insufficiently equipped to accommodate the complexity of MLTC, particularly where single-condition recommendations conflicted or risked harmful interactions between conditions, treatments, or patient priorities. Clinicians therefore articulated needing to adapt, negotiate, and sometimes circumvent formal processes in order to deliver care they perceived as safe and appropriate. These findings resonate with Braithwaite’s description of frontline healthcare work as involving clinicians “muddling through with purpose”, continually finding ways to make imperfect systems function despite uncertainty and constraint [42]. However, while organisational resilience literature highlights the importance of flexibility and adaptive capacity, our findings also highlight participants’ perceptions of the burdens associated with sustained reliance on compensatory practices. The burden of maintaining system function frequently fell upon frontline staff through additional coordination work, emotional labour, and ongoing assessment of risk. Rather than placing responsibility for resilience solely on frontline staff, healthcare systems must be redesigned to better support the realities of MLTC care and reduce reliance on compensatory practices.

### Strengths and limitations of the current study

A key strength of this study is its direct focus on clinical decision-making, offering fresh insights into how HCPs in acute and emergency settings navigate the complexity of delivering health care for people living with MLTC. The study also draws on a large and diverse sample of 40 participants across varied roles, specialties, and locations, capturing both shared challenges and distinct, context-specific perspectives. However, the volume and richness of the interview data also presented a limitation, generating narratives and concepts that could plausibly have been explored in considerably greater depth than was possible within a single paper. In synthesising across accounts, some nuance and powerful illustrative quotations may have been lost. In addition, the study was not initially designed around a formal framework for understanding factors influencing decision-making; exploration of influencing factors was intended to form only one component of the interview schedule. However, these factors emerged as a dominant feature of participants’ accounts. As a result, we applied the SEM framework after data collection, rather than incorporating this framework at the design stage of the study. In hindsight, using a determinant framework from implementation science to inform the interview schedule and subsequent analysis may have provided a clearer and more systematic structure for exploring these influences while also offering a more structured approach to identifying strategies to address them. Nevertheless, the study deliberately sought to explore both the “how” and the “why” of decision-making, which required flexibility beyond a fixed explanatory framework.

While we included a range of professional groups, this study did not capture patient or carer perspectives, which would offer valuable complementary insights into clinical decision-making. However, these perspectives have been explored within the ADMISSION research programme through a recent qualitative study examining the lived experience of inpatient hospital care among people living with multiple long-term conditions (MLTC) [36]. There are clear parallels between the findings of the patient-focused study and our clinician-focused analysis. Both identify persistent tensions between care for acute and chronic conditions within a predominantly specialist model of hospital care, resulting in uncertainty in care pathways. A further disconnect emerges between the complexity of patients’ needs and the organisation of care around single-condition approaches, reflected in the continued reliance on single-condition clinical guidelines [22]. These challenges are navigated within inflexible models of care and resource-constrained hospital settings, shaping experiences of hospital care for people living with MLTC and clinicians’ capacity to make holistic decisions. Across both studies, integrated care, effective inter- and intradepartmental communication, and generalist clinical knowledge emerge as important in supporting holistic and efficient clinical decision-making and care within hospital systems.

Finally, we acknowledge that, in the reporting of this qualitative study, the language used is heavily biomedical, which may appear at odds with the emphasis on experiential perspectives in qualitative health research. However, this reflects the focus of the study on clinicians’ decision-making. Clinicians necessarily draw on biomedical concepts and terminology when describing and enacting decision-making in acute care settings, and the findings therefore inevitably reflect this orientation. This biomedical framing does not exclude broader psychosocial considerations, which are also evident within participants’ accounts and are reflected in the analysis and discussion.

### Directions for future research

Future research should explore how the strategies identified here can be implemented in practice, recognising that improving care for people living with MLTC is unlikely to be achieved through new pathways or recommendations alone. Interventions must account for the complexity and adaptive nature of frontline clinical work and their interaction with existing workflows, workload pressures, communication structures, and team dynamics. Simulation-based approaches, and implementation studies, alongside ethnographic and observational methods, may be particularly valuable for understanding how proposed changes influence decision-making and care delivery in practice.

As many barriers to effective decision-making appear rooted in healthcare system organisation rather than individual clinical practice, future work should involve policymakers, commissioners, service leaders, clinicians, and people living with MLTC. Co-produced approaches, supported by embedded research and process evaluation, may help translate these findings into sustainable system-level improvements.

### Implications for practice, policy, or theory

Drawing on our findings, we identify five recommendations for improving alignment between care delivery and the needs of people living with MLTC (Box 1). Consistent with the SEM framing of our analysis, these recommendations span individual, interpersonal and organisational and system levels.

First, strengthen intermediate care and community support. Clinicians described how many of the blockages they encountered stemmed from limited awareness of, or restricted access to, community-based services. This often made them hesitant to discharge patients without confidence that appropriate safety nets were in place. Addressing this may require both increasing community capacity and improving clinicians’ awareness of available services through clearer referral pathways, better directory systems, and stronger links between acute and community teams.

Second, in the absence of dedicated MLTC pathways or specialist roles, clinicians highlighted the value of expanding generalist, frailty, and geriatric medicine expertise within the care pathway. HCPs working in geriatric medicine and frailty teams described managing MLTC and complexity as routine, reflecting specific training and experience in this area. The use of Comprehensive Geriatric Assessment enabled a more holistic consideration of patients’ medical, functional, and social circumstances, and was associated with greater comfort in working with uncertainty. Facilitating decision-making for people living with MLTC could draw on models of holistic, uncertainty-tolerant practice and extend their use across other teams and specialties. Alongside workforce expansion, this might involve strengthening training in generalist assessment, MLTC management and complexity-informed decision-making across specialties.

Third, participants emphasised the importance of supporting relational, multidisciplinary decision-making including creating the time, protected opportunities and structures for teams to collaborate in navigating uncertainty.

Fourth, clinicians described how time-based targets can work against good care for people living with MLTC. Rather than applying a uniform approach, there may be value in removing these targets and exploring greater flexibility for people living with MLTC, recognising that complexity can require different care pathways and models of care and assessment.

Finally, there is a need to improve continuity of care and shared clinical ownership for people living with MLTC. Participants described how responsibility for patients often shifted between specialties and services, creating uncertainty about who was coordinating care and increasing the risk of fragmented decision-making. Improving continuity may require clearer allocation of responsibility, better communication across organisational boundaries, and care models that support coordination across the patient journey rather than within individual episodes of care.

These suggestions correspond with recent recommendations from UK health policy think tanks to strengthen care for people living with MLTC. The King’s Fund highlights the need for holistic, coordinated care and clearer responsibilities among those involved in MLTC management [46]. Separately, Re: State proposes establishing a new generalist hospital specialty to provide clinical leadership for patients with undifferentiated, complex needs across hospital and community settings [47]. However, our recommendations come with the recognition that mandating such changes in a top-down fashion may be counterproductive. As work in the domain of complexity science highlights, actors within the health and social care system, including patients and healthcare providers across hospital, specialist, community, and social care settings, must be given the autonomy to respond adaptively to complexity and uncertainty as systems change dynamically over time [48]. Services must do more than co-locate; they must co-evolve – developing and adapting together through sustained collaboration. This should be seen as an iterative process that develops over time rather than as the outcome of a one-off initiative [49].

Box 1: Recommendations for supporting decision-making around people living with MLTC **Strengthen intermediate care and community support**

- Consider how community-based options can be made more visible and accessible to clinicians making time-pressured decisions. This may include increasing community capacity, improving awareness of available services through clear referral pathways, better directory systems and stronger links between acute and community teams.

**Expand generalist, frailty and geriatric expertise more broadly and earlier in pathway**

- Explore ways for this expertise to be available upstream, where it can shape clearer, more holistic plans. This could involve workforce expansion and training in generalist assessment and complexity informed decision-making across clinical disciplines.

**Support relational, multidisciplinary decision-making**

- Create conditions that allow teams to communicate, share responsibility, and make sense of complex situations together for example by embedding collaborative decision-making structures including opportunities for multidisciplinary discussion.
**Reduce reliance on time-based targets for complex patients**
- Reflect on how rigid targets may constrain decision-making for people living with MLTC and where flexibility might be needed recognising that complexity can require different models of care and assessment.
**Improve continuity and shared ownership**
- Consider structures that support smoother transitions across care settings, clearer allocation of responsibility and better communication across teams and settings.

## Conclusions

Clinical decision-making for people living with MLTC emerges through the interaction of factors across patient and professional, interpersonal, and environmental levels, generating persistent uncertainty that clinicians navigate through adaptive strategies including heuristics, risk mitigation, and relational ways of working. While these strategies enable care to continue in complex settings, they also involve substantial invisible work and may inadvertently reproduce the system pressures they seek to manage. Our findings highlight that improving decision-making around referrals, admissions, and care planning for people living with MLTC may benefit from greater alignment between patient need and the organisation of healthcare services, and from reducing reliance on compensatory practices. This will require strengthening relational and multidisciplinary working, expanding generalist expertise, reducing reliance on hospital time-based targets, improving coordination across services, and enhancing intermediate and community-based support to reduce pressure on acute pathways.

## Supplementary information

Below is the link to the electronic supplementary material.

Supplementary Material 1: S1: Project Summary

Supplementary Material 2: S2: Participant information sheet

Supplementary Material 3: S3: Consent form

Supplementary Material 4: S4: Interview schedule

Supplementary Material 5

## Data Availability

Pseudonymised interview data used in this publication which are not provided in the supplementary materials are available from the corresponding author upon request.

## Abbreviations

A&E: Accident and Emergency
ABCD: Airways, Breathing, Circulation, Disability
ACP: Advanced Clinical Practitioner
AI: Artificial Intelligence
CHAIN: Contact, Help, Advice and Information Network
COREQ: Consolidated Criteria for Reporting Qualitative Research
ED: Emergency Department
HCP: Healthcare Professional
HRA: Health Research Authority
HRC DTE: HealthTech Research Centre in Diagnostic and Technology Evaluation
MDT: Multidisciplinary Team
MLTC: Multiple Long Term Conditions
NHS: National Health Service
NIHR: National Institute for Health and Care Research
OT: Occupational Therapy
PPIE: Patient and Public Involvement and Engagement
PAG: Patient Advisory Group
PT: Physiotherapy
RAFT: Rapid assessment, fast treatment
RAP: Rapid Assessment Sheet
REC: Research Ethics Committee
ReSPECT: Recommended Summary Plan for Emergency Care and Treatment
SDEC: Same Day Emergency Care
SEM: Socio Ecological Model
WAD: Work as done
WAI: Work as imagined
