# Supplementary material for: Front-line decision-making: a thematic analysis of interviews with hospital staff on referrals, admissions, and care for people with multiple long-term conditions
Source: BMC Med. 2026 Oct 16;24:568. doi: 10.1186/s12916-026-05236-6 (PMC13673829; doi:10.1186/s12916-026-05236-6)
Supplement: Supplementary file 1 [file 12916_2026_5236_MOESM1_ESM.pdf]

Project Summary

Clinical decision-making and patient pathways for multiple long-term conditions in hospital patients

Multiple long-term conditions (MLTC, also known as multimorbidity) are an escalating healthcare challenge due, in large part, to increasing life expectancy and growing incidence of chronic health conditions. The rising prevalence of MLTC is associated with an increasing number of ED presentations, hospital admissions and hospital bed days occupied.

The NIHR Newcastle HealthTech Research Centre (Newcastle HRC), as part of the ADMISSION Research Collaborative are running a research project to help understand NHS staff perspectives on how patients with MLTC move through the hospital (with a particular focus on pathway choices at the start of hospital stay) and how decisions are made about where patients with MLTC are referred to. Our role as an independent NIHR body is to help generate impartial evidence regarding clinical decision making and management for patients with MLTC within secondary care.

We are looking to interview healthcare professionals involved in decision-making around patient allocation to wards/clinical specialties in the hospital.

Key staff members of interest are:

- NHS hospital staff based in the emergency department (ED), assessment suites and other acute assessment areas from different roles and specialties (clinicians, surgeons, physiotherapists, nurses, microbiologists from specialist/ general wards / critical care, and managers).

Interviews will last between 45 minutes to 1 hour and will be conducted either by Zoom /MS Teams, phone or face to face depending on the participant's preference.

Expressions of interest can be sent to sara.pretorius@newcastle.ac.uk
