# Supplementary material for: Front-line decision-making: a thematic analysis of interviews with hospital staff on referrals, admissions, and care for people with multiple long-term conditions
Source: BMC Med. 2026 Oct 16;24:568. doi: 10.1186/s12916-026-05236-6 (PMC13673829; doi:10.1186/s12916-026-05236-6)
Supplement: Supplementary file 3 [file 12916_2026_5236_MOESM3_ESM.pdf]

Participant Identification Number (PIN):____________

Informed Consent Form

Study: ADMISSION-CPA: Clinical decision-making and patient pathways for multiple long-term conditions in hospital patients. Semi-structured interviews with healthcare professionals.

Senior methodologist: Dr Jana Suklan
Lead methodologist: Dr Sara Pretorius

**All Participants**	**Please initial these boxes to confirm:**
I have read and understood the Participant Information Sheet (V1.0, 12/09/2023) for the above study.	
I have had the opportunity to consider the information, ask questions and have had these questions answered satisfactorily.	
I understand that my participation is voluntary and that I am free to withdraw at any time, without giving a reason and without my legal rights being affected.	
I understand that I can withdraw permission to use data from my interview up until the point it had been analysed.	
I accept that the researchers take no liability for any accidental breach of patient confidentiality by myself during the interview. I understand that this information will be omitted from any output arising from the interview, but that an audio recording (if consented to, below) may remain.	
I understand that my details, interview recording and/or associated documents may be accessed by authorised individuals of the sponsor team for the purposes of audit.	
I give permission, for the publication of anonymised quotes.	
**I agree to participate in the above titled study.**	

**Optional**	**Please initial if you agree**
I give my consent for the conducted interview to be audio and/or video recorded and stored securely for a period of five years.	
I understand that I may be contacted after the interview, if required, for clarification of key points.	
I am willing to be contacted to take part in future research and give permission for my contact details (name and email address) to be stored on a secured database for this purpose.	

______________________	______________________	______________
Name of Participant	Signature	Date (dd.mm.yyyy)

Once completed ensure one copy for the participant, one copy to be retained
