# Supplementary material for: Front-line decision-making: a thematic analysis of interviews with hospital staff on referrals, admissions, and care for people with multiple long-term conditions
Source: BMC Med. 2026 Oct 16;24:568. doi: 10.1186/s12916-026-05236-6 (PMC13673829; doi:10.1186/s12916-026-05236-6)
Supplement: Supplementary file 4 [file 12916_2026_5236_MOESM4_ESM.pdf]

ADMISSION-CPA: Clinical decision-making and patient pathways for multiple long-term conditions in hospital patients

Participant Information Sheet

Summary

The aim of this study is to improved understanding of how NHS staff make decisions regarding patients with multiple long-term conditions (MLTC). If you agree to take part, you will be interviewed about how you make decisions on where patients are referred to, who looks after them, and where they are looked after.

In this research study we will use information that you will provide to us in the interview. We will only use information that we need for the research study. We will let very few people know your name or contact details, and only if they really need it for this study.

Everyone involved in this study will keep your data safe and secure. We will also follow all privacy rules.

At the end of the study we will save some of the data in case we need to check it.

We will make sure no-one can work out who you are from the reports we write.

1 Introduction

The following information is provided to help you decide whether you want to take part in this study. It is important that you understand why the research is being done and what it will involve. Please take the time to read this sheet carefully, but also please feel free to contact a member of the research team (details below) if there is anything about the study you do not understand or if you would like more information.

What is the study about?

A growing number of patients presenting to hospital now have Multiple Long-term Conditions (MLTC) – defined as two or more long-term health conditions. We know that hospital systems are not currently set up to provide care for this group of patients, and patients with MLTC report lower satisfaction with care.

To create better healthcare systems for patients living with MLTC, we need to understand what the current pathways of care for this group of patients are when they present to hospital – what happens, what works well and what does not work well. Part of this work is to understand the perspectives of clinicians looking after patients with MLTC, what decisions clinicians make regarding care for these patients when they present to hospital, and what drives these choices. In this study, we are particularly trying to understand choices around where patients are referred to, who looks after them, and where they are looked after.

Who are we looking for?

We are seeking healthcare professionals, with experience in making decisions around care of patients with MLTC. We want to explore your perspectives regarding how these patients move through the hospital with a particular focus on pathway choices at the start of the hospital stay.

What does the study involve?

As a participant of this study, you will be invited to take part in a semi-structured interview on decision-making around patient allocation to wards/clinical specialities in the hospital. The interview will be conducted by video conferencing (Teams or Zoom), by telephone or face to face if you prefer, on a mutually agreed time and date and will last between 45 to 60 minutes. At the beginning of the interview, the researcher(s) will explain the purpose and format of the interview, including the types of questions that will be asked. We will be using a series of 'vignettes'. Each vignette is a short account of a patient presenting with MLTC at the emergency department/acute assessment suite and gives details including their clinical history, medication and presenting symptoms. The vignettes will be discussed during the interview to explore factors in your decision-making in various clinical scenarios.

Prior to the interview, you will be sent a copy of the vignettes and a consent form electronically. The consent form will outline the ethical issues associated with the study. One of the ethical considerations of this study is whether you are willing to have the interview recorded. Recording is not mandatory for participation, but is preferable, as it will ensure that we can fully recall the information you provide. All transcribed data will be fully anonymised and no identifiable information will be linked to the use of these audio recordings. No personal information will be shared at any stage of this research. Other ethical considerations relate to the secure storage of data; for information on these please see the section titled 'Personal information policy', below. If, having agreed to the terms in the consent form, you are happy to proceed with the interview, you will be asked to sign and return the consent form **before the scheduled interview**. Verbal consent can be obtained online at the start of the interview session if the form cannot be completed prior to the interview.

On the consent form, you are given the option of agreeing to be contacted after the interview, if required, for clarification of key points. If you consent to follow-up contact, you may be contacted for further explanation or clarification of a response given during the interview. This should require a limited time commitment (e.g. 5-10 minutes for an email response or short conversation). We attempt, where possible to avoid follow-up contact by ensuring that key points are comprehensively explored during the interview and you may choose not to consent to follow-up contact if you prefer. You are also given the option to agree for your name and email address to be stored on a secure database so that you may be contacted regarding participation in future research. The database will be kept securely on a password-protected computer, in an encrypted folder, at Newcastle University. It will be reviewed on an annual basis at which time you will be contacted and asked whether you wish for your details to remain on the database or for them to be removed. You may contact us at any time to request that your name and contact details be removed from the database.

Do I have to take part?

It is completely up to you whether or not you take part in this study; your participation is voluntary. You may leave the study at any point without giving a reason.

What are the possible benefits of taking part?

The study itself will not benefit you directly, but the information that we will gain from your participation may lead to changes in healthcare services arising from a better understanding of current clinical practice for managing patients with multiple long-term conditions and the potential challenges of making decisions around the clinical care of MLTC patients. We will share a summary of the study results with participants at the end of the study.

What are the possible risks of taking part?

We do not anticipate any potential risks associated with taking part in this study.

Personal information policy

1.1 Study Sponsor

The organisation sponsoring this study is Newcastle University, based in the United Kingdom see https://www.ncl.ac.uk/research/researchgovernance/ethics/ethicstoolkit/sponsorship/ for details). Any references to 'we' in this document will refer to Newcastle University on behalf of the research team.

How we will use your information

We will need to use information from you for this research project. This information will include:

- Your name
- Your email address
- The data that you provide us with during the interview

People will use this information to do the research or to check your records to make sure that the research is being done properly. We will keep all information about you safe and secure. Once we have finished the study, we will keep some of the data so we can check the results. We will write our reports in a way that no-one can work out that you took part in the study.

Newcastle University will act as data controller for this study. This means that we are responsible for looking after your information and using it properly. The organisation will keep identifiable information about you for 10 years after the study has finished. More specifically, all personal data collected from participants (including name and contact details) will be kept confidential, conforming with the rules of the General Data Protection Regulations 2018 (GDPR), stored securely on a password-protected computer, in an encrypted folder, at Newcastle University for a period of 10 years. Personal data will only be accessed by the team of researchers working on this project, named at the end of this form. People who do not need to know who you are will not be able to see your name or contact details. Your data will have a code number instead. No personal information will be shared at any point of the research.
What are your choices about how your information is used? You can stop being part of the study at any time, without giving a reason, but we will keep information about you that we already have. You can withdraw your data up until the point it has been analysed (up to two weeks after the interview has been conducted). If you agree to take part in this study via an option in the consent form, you will have the

option to be contacted again to take part in future research using your data saved from this study . You can change your decision on additional contact at any point by contacting the research team (see contact details below).

1.2 Finding out about how your information is used

You can find out more about how we use your information

- by sending an email to nihr.newcastle.mic@newcastle.ac.uk , or
- by ringing us on (0)191 2083709.

If you have any further questions regarding your information, please contact one of the researchers listed at the end of this document or alternatively email Newcastle University's Data Protection Officer directly at rec-man@ncl.ac.uk.

1.3 Data Protection Policy

All of our research must comply with the General Data Protection Regulation 2018 (GDPR) rules and governed by the Data Protection Act. Newcastle University's data protection policy can be found at:

https://www.ncl.ac.uk/data-protection/data-protection-policy/
GDPR Information for Research can be found at:
https://www.ncl.ac.uk/research/researchgovernance/ethics/gdpr/

Funding

The NIHR Newcastle In Vitro Diagnostics Co-operative has been funded by UKRI-NIHR to conduct this study as part of the ADMISSION Research Collaborative.

How do I get involved?

To participate in this study, or if you have any questions, please contact Dr Sara Pretorius at sara.pretorius@newcastle.ac.uk.

Research Team

Professor Miles Witham miles.witham@newcastle.ac.uk
Dr Jana Suklan jana.suklan@newcastle.ac.uk
Dr Sara Pretorius sara.pretorius@newcastle.ac.uk
Dr Nicola Howe nicola.howe@newcastle.ac.uk
